# Exploring the genetic alterations of Gorham-Stout disease

**DOI:** 10.3389/fendo.2025.1654497

**Published:** 2025-08-19

**Authors:** Olivia Pagliarosi, Jessica Pepe, Andrea Del Fattore, Michela Rossi

**Affiliations:** ^1^ Department of Clinical, Internal, Anesthesiology and Cardiovascular Sciences, Sapienza University, Rome, Italy; ^2^ Bone Physiopathology Research Unit, Translational Pediatric and Clinical Genetic Research Division, Bambino Gesù Children’s Hospital, IRCCS, Rome, Italy

**Keywords:** Gorham-Stout disease, rare disease, genetic characterization, genetic variants, molecular pathways

## Abstract

The “vanishing bone disease” or Gorham-Stout disease (GSD) is a very rare disorder characterized by massive lymphatic and angiomatous proliferation accompanied by progressive osteolysis, without the deposition of new bone matrix. Because of its rare and complex clinical features, diagnosis is challenging and its etiopathogenesis is not completely known; the genetic basis of GSD has been hypothesized and different mutations have been reported in patients. Our review aims to describe all these genetic alterations found in GSD patients and their association with clinical features. The identification of a specific molecular pathway or genetic alteration in GSD could help in the diagnosis and possibly the treatment of this rare sporadic disease.

## 1 Introduction

Gorham-Stout disease (GSD), also known as “vanishing bone disease”, is a very rare disorder, to date not even 400 cases have been described, and is characterized by lymphatic and angiomatous proliferation accompanied by progressive osteolysis. Although a slight predilection for male, the disease does not show a clear sex bias or inheritance pattern and can occur at any age, with the majority of cases occurring in childhood (1), affecting one or multiple bones of either the axial or the appendicular skeleton; patients initially display a patchy osteoporosis condition, which progressively leads to skeletal deformity, shrinkage and eventual loss of the affected bone (2, 3). Moreover, both the medullary and cortical regions of affected bones present lymphatic vessels, which are not typically found in normal bones (4). The main reported symptoms are pain, weakness and impairment of the affected regions; however, some patients develop more severe complications such as chylothorax (5), which may cause respiratory distress, as well as vertebrae involvement leading to neurological deficits or paraplegia, bone infection and subsequent septic shock, and ultimately death (2).

**Figure 1:**
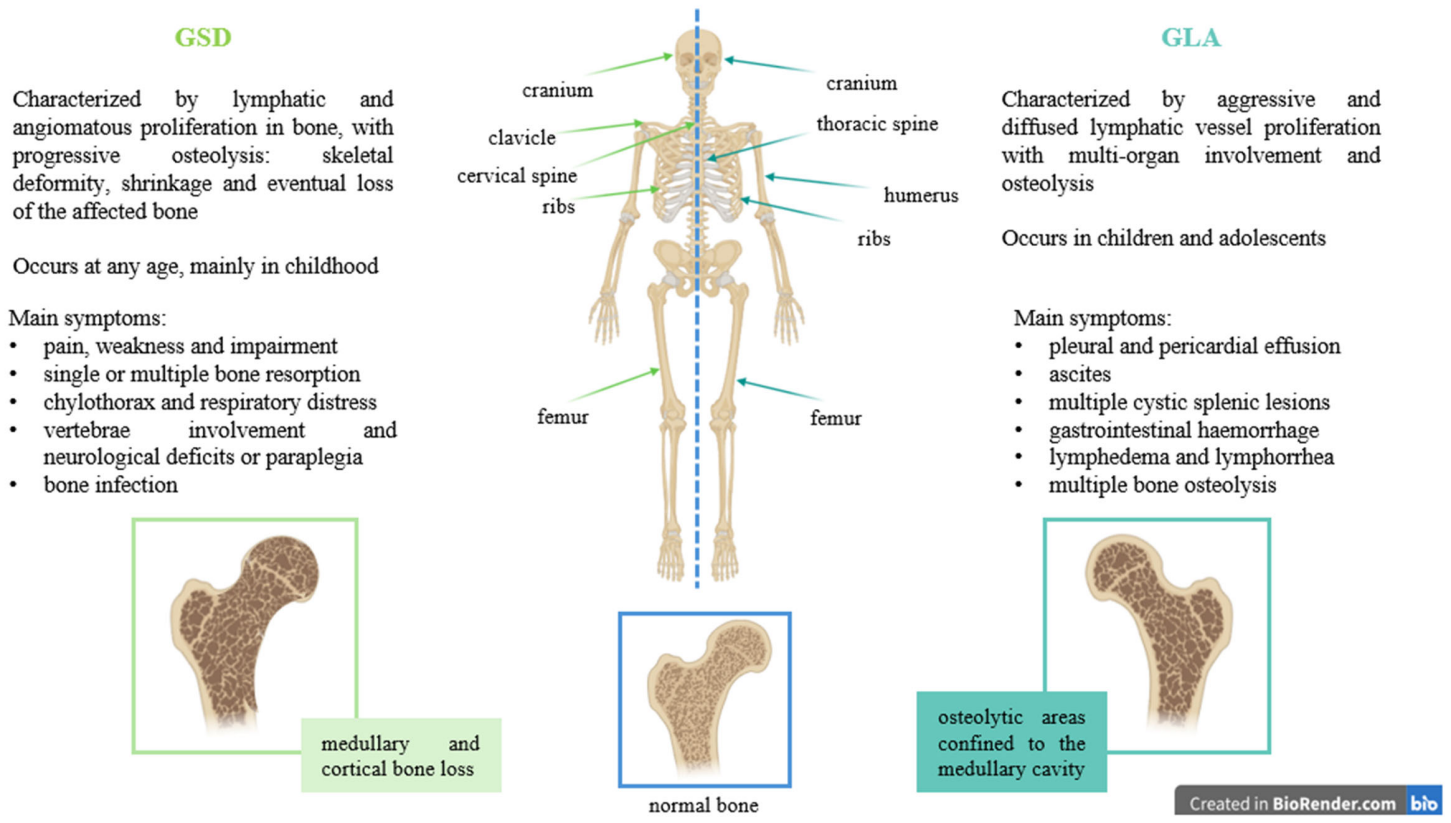
Differences between GSD and GLA, highlighting symptoms, bone involvement and type of osteolysis.

GSD is frequently undiagnosed or misdiagnosed and is indeed often classified as a complex lymphatic abnormality (CLA) as some clinical features overlap with CLA diseases, such as generalized lymphatic anomaly (GLA), Kaposiform lymphangiomatosis (6) and channel-type lymphatic malformations (LM) (2). GLA is a rare and aggressive disease characterized by diffused lymphatic vessel proliferation with multi-organ involvement (mediastinum, lungs, bone, spleen, and soft tissues), and it primarily affects children and adolescents. Its symptoms include pleural and pericardial effusion, ascites, multiple cystic splenic lesions, gastrointestinal haemorrhage, multiple bone osteolysis (mostly skull and spine), lymphedema, and lymphorrhea (7) (**Figure 1**). GSD, in contrast, is distinguished by the progressive destruction and erosion of bone, particularly the cortical bone, often leading to its complete absorption with the presence of abnormal intraosseous LM, found also in regions adjacent to osteolytic lesions (**Figure 1**). Hu et al. carried out radiographic evaluations of 67 GSD cases, half of which present the disappearance of portions of bone; the initial stage of this process manifests as radiolucent foci in the intramedullary or subcortical regions (8). The femur was the most commonly involved site (8). In the same year, Lala et al. carried out another radiological study on a cohort of 51 patients, of which 19 met the criteria for GSD, and highlighted differences in osteolytic activity between GLA and GSD patients: GLA generally shows lytic areas confined to the medullary cavity, whereas GSD displays progressive osteolysis, with cortical bone loss (9). They also identified the ribs as the most commonly affected site in both groups, followed by the cranium, clavicle, and cervical spine in GSD, while the thoracic spine, humerus, and femur were most frequent in GLA. Additionally, GSD typically involves fewer bones than GLA (9) (**Figure 1**). Further investigation is necessary to fully differentiate these two lymphatic disorders.

Therefore, the diagnosis of GSD remains challenging with no standardized guidelines; it is usually performed by exclusion criteria to rule out neoplastic processes, infections, as well as metabolic and endocrine disorders. Radiographs, bone scan and computed tomography are useful (10), but diagnosis must be confirmed by histopathological analysis of the bone lesion, which should reveal extensive bone resorption and angiomatous tissue, as well as excessive presence of fibrotic tissue without cellular atypia (11, 12). Indeed, the osteolytic process is characterized by the absence of increased osteoblast activity along the surfaces of the remaining bone fragments in sections of affected tissues; the disappearing bone is replaced by fibrovascular tissue rather than newly formed woven repair bone (2). Moreover, GSD osteoblastic cells exhibit ultrastructural alterations suggesting that they have either decreased synthetic activity or are undergoing degeneration (13). Furthermore, osteocytes within bone tissue close to the lesions have been reported to present enlarged lacuna, possibly related to osteocyte mediated-bone resorption activity (2, 12).

Due to its rarity and limited case studies, also the etiopathogenesis of GSD remains unclear. It has been proposed that the osteolytic process may result from excessive proliferation of endothelial and lymphatic vessels, accompanied by increased local blood flow, changes in pH or altered mechanical forces that affect bone remodeling (2). The secretion of cytokines and growth factors such as TNF (Tumor Necrosis Factor)-α, IL (Interleukin)-6 and VEGF (Vascular Endothelial Growth factor)-A/C has also been considered, given their stimulatory effect on lymphangiogenesis and osteoclast activity (7, 14–16), and their inhibitory effect on osteoblasts, particularly at high concentrations (2, 17–19). Recent studies have proposed that GSD may involve a primary imbalance in bone remodeling, characterized by increased osteoclast differentiation and resorption (20), along with an impairment of circulating bone cells (21). Moreover, immune dysregulation or inflammatory conditions could contribute to exacerbate the syndrome (22).

Depending on the severity of the disease and the extent of organ involvement, different strategies are used to treat GSD symptoms, including surgery, radiotherapy and drug treatment, which are only partially effective and none are curative, but patients experience relief and improved quality of life after treatment (1). Bone loss with functional impairment requires surgical procedures which consist of the resection of localized lesions and the reconstruction or stabilization of the bone by using bone grafts and/or prostheses (8, 23, 24); interventions are also carried out to prevent respiratory insufficiency and reduce or halt the fluid build-up in the pleural cavity (24). Without surgical intervention, morbidity and mortality rates are very high (2).

The use of radiotherapy has been described as successful and beneficial in several case reports, with a 75% overall success rate on local lesions with doses in the 30–45 Gy range (25–27). It has also been used to manage chylothorax in a GSD case (28). However, radiation could provoke serious problems, like secondary malignancy and growth restriction in children and adolescents who receive a high dose treatment (29).

Bisphosphonates and interferon alpha 2b are commonly prescribed. Bisphosphonates have been successfully used for the treatment of GSD patients for their anti-osteoclastic activity (30, 31). Hammer et al. reported a stabilization of the clinical and radiological picture in a patient treated with pamidronate every 3 months (31). In 2014, Liu et al. evaluated different bisphosphonate treatments (either zoledronic acid or pamidronate) with or without radiotherapy (40 Gy) on GSD patients, all of which stabilized disease progression as well as inhibited the enlargement of the osteolytic scope and increased bone mineral density (30). The authors also reviewed GSD bisphosphonate treatment investigations in literature, finding that the most commonly used was zoledronic acid, followed by alendronate, which often led to disease arrest (30). However, the use of anti-resorption drugs like bisphosphonates is often associated with side-effects, including atrial fibrillation, osteonecrosis of the jaw (1) and “frozen bone”, characterized by over-suppression of bone turnover (32). Interferon alpha 2b, an immunomodulatory and anti-angiogenic compound, has been used to stabilize GSD and can also be used in combination with bisphosphonates or after surgery (33–36). Other pharmaceuticals administered for clinical relief are the anti-angiogenic VEGF-neutralizing antibody Bevacizumab (37), propranolol (38), low molecular weight heparin (39), steroids, and vitamin D (2).

More recently, the repurposing of oncogenic treatments, such as Sirolimus or Alectinib, has become more frequent and will be further described in this review.

Over the past few years, the genetic basis of GSD has been largely hypothesized in case reports, although no definitive evidence has yet been established. The identification of specific germline mutations or somatic mosaicism in GSD could significantly aid in the diagnosis and possibly the treatment of this rare condition. This review aims to summarize all the reports investigating genetic alterations in GSD patients and explore their potential contribution to disease development, paving the way for the discovery of new therapeutic targets.

## 2 Genetic studies

In 2013, the first work suggesting that GLA and GSD patients could carry abnormal genomic copy number was presented at the first International Conference on Generalized Lymphatic Anomaly and Gorham-Stout Syndrome (11). Although no updates or reports have been released on that study, other genetic studies have emerged (**Table 1**).

**Table 1:** Summary of the genetic variants identified in GSD patients.

Refs	Gene and role of the protein	HGVS DNA	HGVS Protein	GSD patient clinical features	*In silico* prediction	Associated diseases
(42)	*PTEN*	c.517C>T	p.Arg173Cys	Absence of VIIIth and IXth rib on the right side Extensive pleural fluid Osteolytic lesions of humerus, femur, and tibia	Pathogenic	Macrocephaly-autism syndrome, Cowden syndrome 1, PTEN hamartoma tumor syndrome, Malignant lymphoma, large B-cell, diffuse
(62)	*EML4::ALK*	intron 19 in *ALK* intron 2 in *EML4*	n/a	Diffuse lytic changes and extensive lymphatic malformation throughout the lumbar spine and pelvis	Constitutively active chimeric protein	n/a
(71)	*GATA2*	c.379C>A	p.His127Asn	Cardiac tamponade Lytic lesions in the cervical and thoracic vertebrae Fracture of T4 vertebra	Uncertain significance	n/a
(77) (87)	*KRAS*	c.182A>G c.35G>T	p.Gln61Arg p.Gly12Val	Osteolysis of the right maxilla and of skull base Lytic lesions of right clavicle, humerus, radius, cubitus, right femur, tibia and the D10 vertebra	*Gain*-*of*-*function* *Gain*-*of*-*function*	n/a Non-small cell lung carcinoma RASopathy Chronic myelogenous leukemia, BCR-ABL1+
(89)	*GSDMD*	c.823G>C	p.Asp275His	Osteolysis of the distal phalanx of left thumb and fourth, second and third digits	Missense	n/a
(97)	*TNFRSF11A*	c.1070C > T	p.Thr357Ile	Osteolysis of left scapula and the 7th–9th left ribs and pleural effusion	Benign	n/a
(96)	*TNFRSF10A*	n/a	n/a	Osteolysis of the left humerus associated with vascular proliferation	n/a	n/a
	*PIK3AP1*	c.1139A>T	p.Glu380Val		Deleterious	
	*ATG101::SLC4A8*	intron 3 in *ATG101* intron 1 in *SLC4A8*	n/a		n/a	
	*SGCD::DNAH11*	n/a	n/a		n/a	

n/a, not available.

*In silico* prediction analysis and associated diseases identified by Mutation tester (genecascade.org/mutationtester2025).

### 2.1 PTEN

In 2012, Hopman et al. described a patient exhibiting absence of the 8th and 9th ribs on the right side, extensive pleural fluid, as well as osteolytic lesions in the humerus, femur and tibia. Additionally, he presented a vascular tumor affecting part of the right axilla and flank (42). Diagnosed with GSD and PTEN hamartoma tumor syndrome, the patient underwent genetic analysis which identified two variants in lymphocyte–derived DNA: a germline heterozygous mutation c.517C>T (p.Arg173Cys) of the PTEN (Phosphatase And Tensin Homolog) gene and a polymorphism c.649-26G>T in the TSC2 (TSC Complex Subunit 2) gene. Moreover, the analysis of DNA from the affected tissue revealed also the heterozygous variant c.2180C>T (p.Ala727Val) in the FLT4 (Fms Related Receptor Tyrosine Kinase 4, also known as VEGFR3) gene (42).

The TSC2 polymorphism was deemed non-pathogenic, while the PTEN mutation has been already described in patients with hamartoma (43). PTEN mutations have also been implicated in atypical endometrial hyperplasia, endometrial carcinoma (44), and glioblastoma (45, 46), bone metastases (47), multiple myeloma (48, 49), osteosarcoma (50–52), and other bone malignancies (53).

*PTEN* encodes a dual-specificity phosphatase protein that negatively regulates the PI3K/Akt/mTOR and MAPK (mitogen-activated protein kinase) signaling pathways. Stambolic et al. observed that Pten-deficient murine fibroblasts exhibited decreased sensitivity to cell death, as well as elevated protein kinase B or Akt (PKB/Akt) activity and phosphorylation (54), which promotes survival and oncogenesis (55). As a tumor suppressor, PTEN reduces intracellular phosphatidylinositol 3,4,5-trisphosphate levels, thereby inhibiting the PI3K/PKB/Akt axis (53, 54). Somatic changes in the PI3K/AKT/mTOR pathway have been observed in LM (7, 56). PTEN is further implicated in the regulation of osteoclast differentiation, survival and migration, as well as angiogenesis and lymphangiogenesis (57). The involvement of the phosphoinositide 3-kinase (PI3K) pathway in GSD is also supported by Rossi et al., who performed a transcriptomic analysis on mature osteoclasts differentiated from peripheral blood mononuclear cells of GSD patients, revealing an enrichment of this pathway (20). To define the role of PTEN in bone homeostasis and bone strength, Lorenz et al. generated a mouse model of Pten conditional Knock-out (Pten-cKO) in pre-osteoblasts and investigated osteoprogenitor cells; bone marrow stem cells isolated from Pten-cKO animals showed enhanced proliferation and osteogenic differentiation, resulting in increased trabecular bone volume and mechanical strength (58).

The mammalian target of rapamycin (mTOR) is a downstream kinase involved in the PI3K/Akt pathway, involved in metabolism, angiogenesis, cell motility and growth; its dysregulation has been documented in LM (59). Hence, the use of Sirolimus, an inhibitor of mTOR, has been tested on multiple vascular anomalies (60, 61) and on a cohort of 5 patients with GSD (41), demonstrating to be efficacious and well tolerated in these studies.

### 2.2 EML::ALK fusion

A recent paper reported a GSD patient with extensive LM in the lumbar spine and sacrum, with chronic cerebrospinal fluid leak and severe headaches. The patient underwent various unsuccessful treatments (62). Genetic testing using an oncology-focused next-generation sequencing panel on patient’s bone biopsy revealed an EML4::ALK (echinoderm microtubule-associated protein-like 4-anaplastic lymphoma kinase) fusion (62).

There are several types of EML4::ALK fusions, all containing the intracellular tyrosine kinase domain of ALK but differing in the truncation sites of EML4 (63). *In vitro* studies using NIH/3T3 mouse fibroblastic cells expressing different EML4::ALK variants demonstrated that these variants mainly activate the MAPK/ERK and STAT3 signaling pathways, promoting cell proliferation, survival, and invasion (63). The MAPK/ERK signaling pathway is associated with cell proliferation, and the mTOR and STAT3 pathways are associated with cell survival and apoptosis. The EML4::ALK fusion protein upregulates MAPK signaling and activates ERK; moreover, increased expression of STAT3 promotes the activation of mTOR thus inhibiting apoptosis of tumoral cells. Studies indicate that some regions of the EML4 gene induce tumorigenesis, in particular the HELP domain is necessary for the specific activation of RAS, which promotes the upregulation of RAS and the phosphorylation of ERK, inducing cell proliferation (64). These variants have been widely reported in tumors, particularly as the primary pathogenic driver in non-small cell lung cancer (65, 66), and have also been identified in a patient affected with GLA (62).

Anaplastic lymphoma kinase (ALK) is a tyrosine kinase receptor that plays a key role during development and is largely not expressed in most adult tissues; indeed, ALK becomes constitutively active when fused with EML4, driving oncogenic signaling; these fusion proteins are validated targets of tyrosine kinase inhibitors such as crizotinib, alectinib or lorlatinib (67–70). The aforementioned GSD patient was treated with alectinib, resulting in reduced swelling and pain, as well as decreased soft tissue edema in the LM (40).

An in-depth investigation into the influence of the chimeric mutation in GSD bone phenotype is still necessary, as well as the analysis of the effects of ALK inhibitors in the progression of the bone disease.

### 2.3 GATA2

In a very short case report, Oguz and colleagues investigated the genomic signature of a pediatric female diagnosed with GSD and presenting cardiac tamponade, who was successfully treated with Sirolimus (71). Genetic analysis was performed using a vascular anomaly panel which includes the NRAS, KRAS, FOXC2, FLT4, GJC2, VEGFC, PIEZO1 and GATA2 genes and heterozygous splicing mutation c.379C>A (p.His127Asn) in the GATA2 (Endothelial Transcription Factor GATA2) gene was detected (71).

GATA2 is a key factor in the generation and maintenance of hematopoietic stem and multipotent progenitor cells, and is involved in hematopoietic diseases, infections and cancer. Its altered expression is associated with immunodeficiency (so-called GATA2 deficiency) and acute myeloid leukemia (72). In hematopoietic stem progenitor cells, GATA2 activates the expression of diverse genes, including those encoding c-Kit receptor tyrosine kinase, erythroid and the megakaryocytic differentiation inducer GATA1 (72). Moreover, it is crucial for lymphatic vessel development (73) as demonstrated in Gata2 heterozygous deficient mice, which display delayed lymphatic recanalization after resection (74, 75). Furthermore, GATA2 modulates the expression of microRNA-126, regulator of lymphatic vessel development (73).

Tolkachov et al. analyzed the deletion of GATA2 in mesenchymal stem cells, revealing increased osteogenic differentiation and bone formation (76). Loss of GATA2 also reduced osteoprotegerin expression, enhancing osteoclastogenesis. *In vivo*, this resulted in enhanced bone formation accompanied by impaired trabecular bone and mechanical strength, confirming a role of GATA2 in bone turnover (76).

### 2.4 KRAS

So far, the most representative genetic study was performed by Nozawa and colleagues who analyzed a cohort of 6 GSD patients. Targeted sequencing analysis of cancer-related genes revealed the somatic KRAS c.182A>G (p.Gln61Arg) variant in frozen affected tissue of one patient (77). This gain-of-function mutation is well-documented in various human cancers (78) and congenital syndromes known as RASopathies (79). The mutation significantly promotes cell growth through the activation of the MAPK and PI3K/AKT signaling pathways (80). Indeed, the KRAS gene encodes K-Ras, a small GTPase that acts as an oncogenic molecular switch, regulating cell proliferation and survival (78, 81, 82). KRAS mutations in CLA patients impair GTP hydrolysis, resulting in hyperactive downstream signaling (78). Cells with oncogenic KRAS variants have an impact on the production of angiogenic and osteoclastogenic cytokines, including VEGF (83) and IL-6 (84). In addition, KRAS-mutant cancer cells modulate the inflammatory response, recruiting and activating immune cells, promoting pro-tumorigenic properties and cell evasion from immunosurveillance; moreover, these cells secrete molecules that promote the recruitment of activated macrophages, which also promote angiogenesis and osteoclastogenesis, contributing to the secretion of VEGF, IL-6 and TNF-α (85, 86). Indeed, Nozawa et al. suggested that macrophages harboring the variant may contribute to the production of large amounts of angiogenic and osteoclastogenic molecules (77).

In 2021, Homayun-Sepehr et al. identified another activating somatic mutation (c.35G>T, p.Gly12Val) of KRAS in the blood and affected tissue of a GSD patient using a comprehensive cancer panel containing 408 cancer-related genes (87). The mutation p.G12V was previously reported in a patient with malignant giant cell tumor of bone, a rare aggressive sarcoma characterized by the presence of multinucleated giant cells and poor clinical course (88). To assess the impact of the hyperactive KRAS variant in the lymphatic system, a genetically engineered mouse model that conditionally expressed the hyperactive form of Kras (p.Gly12Asp) in Lymphatic Endothelial Cells (LEC) was generated. The authors reported growth of lymphatic vessels in bone, impairment in lymphatic valve formation, and the development of chylothorax, resembling the vascular features presented by GSD patients (87). Moreover, a gene ontology analysis of the modulated genes identified in LEC derived from the hyperactive-KRAS mouse model showed increased expression of genes involved in angio/lymphangiogenesis, cell proliferation and migration, and metallopeptidase activity. The role of KRAS mutations in GSD bone still needs to be investigated.

### 2.5 Gasdermin D

More recently, Uehara et al. identified the biallelic missense variant c.823G>C (p.Asp275His) of the GSDMD (Gasdermin D) gene in a GSD patient with osteolysis of the distal phalanx of the left 4th, right 2nd, and 3rd digits, without lymphangiomatous proliferation observed in bone biopsy (89). As the variant is located at the exon-intron splice junction of exon 7, the authors hypothesized that the splicing process could be altered, but no alterations of GSDMD expression were found in a lymphoblastoid cell line derived from the patient’s PBMC (89).

GSDMD is a key regulator of pyroptosis, a type of programmed inflammatory cell death triggered by invasive infection and danger signals. Gasdermins mediate pore formation in the plasma membrane, leading to the loss of cell membrane integrity and leakage of cell cytosolic contents, inducing inflammation (90); GSDMD is transcriptionally regulated by NF-κB and the interferon regulatory factor 2 (91), and its activity is mediated by caspases and the NLRP3 inflammasome, already implied in osteoporosis (92, 93). Upon activation, caspases cleave GSDMD to generate an N-terminal cleavage product that triggers pyroptosis and the release of inflammatory cytokines such as IL-1β (94, 95).

Indeed, the connective tissue surrounding the bone of the aforementioned GSD patient had mild inflammatory cell infiltration, mainly characterized by macrophages. Moreover, investigation of GSDMD cleavage in monocytes of the GSD patient did not reveal the fragments generated with protein activation (89).

GSDMD has already been recognized as a critical player in bone metabolism by preventing bone loss. Indeed, an up-regulation of Gsdmd expression was detected during osteoclast differentiation of the murine macrophage RAW264.7 cell line and mouse bone marrow cells (89). In the late stage of osteoclast lineage commitment, Li et al. observed that the cleavage of Gsdmd also yielded a non-pyroptotic 20-kDa fragment that inhibits excessive osteoclastic resorption and bone loss by restricting the maturation and secretion of lysosomes (91). In fact, Gsdmd-deficient osteoclasts displayed enhanced lysosomal number, size, density and activity, and increased bone resorption activity *in vitro*. The Gsdmd-KO mice displayed an osteoporotic phenotype, with reduced trabecular bone and number, enhanced eroded surface and increased levels of serum bone resorption marker CTX-I (Carboxy-terminal type I collagen) compared to wild-type mice. Osteoblast parameters were minimally affected, indicating that the phenotype was not a result of impaired bone formation. In fact, gene expression analysis further confirmed that the most significant alterations occurred in osteoclasts rather than osteoblasts (91).

### 2.6 Multi-omics analysis

Yebenes Mayordomo et al. recently carried out a multi-omic analysis of data from whole-genome and RNA sequencing of the affected tissue and the surrounding normal tissue in a 45-year-old Gorham-Stout patient (96). A total of 643 mutations were identified across 233 genes, with a high frequency of insertions and deletions; among the most frequently mutated genes, TNFRSF10A, a tumor necrosis factor receptor involved in the mediation of apoptosis and the activation of NF-κB pathway, was identified (96). It belongs to the same receptor family as TNFRSF11A, found mutated (c.1070C > T, p.Thr357Ile) in a patient with GSD presenting osteolysis of the left scapula and the 7th–9th left ribs (97). Although the effect of this variant remains to be functionally characterized, it is well established that TNFRSF11A plays a key role in osteoclast differentiation and activity, and alterations in this gene have been associated with osteolytic diseases (98, 99). Additionally, Yebenes Mayordomo et al. identified a missense mutation (c.1139A>T) in the PIK3AP1 (Phosphoinositide-3-Kinase Adaptor Protein 1) gene (96), involved in the PTEN/PI3K/AKT signaling pathway and known for its role in regulating inflammation and the innate immune response.

The in-depth analysis discovered the presence of a substantial number of interchromosomal mutations, in particular chromosome translocations, suggesting that gene fusion variants could be frequent in GSD (96). The authors noted a gene variant found in chromosome 12 consisting of the fusion of ATG101 (Autophagy Related 101), involved in macroautophagy (100, 101), and SLC4A8 (Solute Carrier family 4 member 8), which could possibly affect the macrophage signaling pathway (96). Another gene fusion involved the SGCD (Sarcoglycan Delta) and DNAH11 (Dynein Axonemal Heavy Chain 11) genes (96); SGCD is associated with muscular dystrophy (102, 103), which could determine vascular malformations (104). These fusion variants still need to be fully described.

A high proportion of genes were either up- or down-regulated and the expression of gene families like VEGF or NOTCH drastically increased in GSD affected tissue compared to normal tissue (96). The expression of PI3K, as well as AKT and mTOR, was detected to be considerably decreased, while PTEN was increased (96), suggesting an altered stimulation of endothelial cell growth and angiogenesis through VEGFA and VEGFB and the VEGFR1-PI3K-AKT signaling pathway (105, 106).

## 3 Conclusions

Gorham-Stout disease is still an enigma. To date, only a few research studies have investigated the genetic alterations in a limited number of patients, employing heterogeneous methods and analyzing various types of tissues.

Many studies reported mutations that affect the PI3K/AKT signaling cascade as well as the MAPK pathway, with several collateral effects on macrophages, which impact osteoclastogenesis and bone turnover; moreover, for some of these pathways, drug treatment is still available (**Table 2**). A comprehensive analysis of these molecular mechanisms, combined with detailed phenotyping of bone involvement in a larger cohort of patients is essential to achieve a more complete understanding of GSD. Such insights are crucial to improve diagnosis and identify new therapeutic targets for this rare and debilitating syndrome.

**Table 2:** Pathways involved with the mutations identified in GSD patients and related available treatments.

Gene	Pathways involved	Available drugs
*PTEN*	Antagonist of the PI3K/Akt/mTOR and MAPK pathways	Sirolimus (inhibitor of mTOR)
*EML4::ALK*	Activates the Ras/MAPK/ERK and STAT3/mTOR signaling pathways	Alectinib (tyrosine kinase inhibitor)
*GATA2*	Maintains hematopoietic stem and multipotent progenitor cells	n/a
*KRAS*	Activates the MAPK and the PI3K/AKT signaling pathways	n/a
*GSDMD*	Mediates pyroptosis and the release of inflammatory cytokines	n/a
*TNFRSF11A*	Activates of NF-κB and MAPK8/JNK pathways	n/a
*TNFRSF10A*	Mediates apoptosis and the activation of NF-κB	n/a
*PIK3AP1*	Activates the PI3K/Akt/mTOR	n/a
*ATG101::SLC4A8*	n/a	n/a
*SGCD::DNAH11*	n/a	n/a
